# Characteristic Volatile Composition of Seven Seaweeds from the Yellow Sea of China

**DOI:** 10.3390/md19040192

**Published:** 2021-03-29

**Authors:** Pengrui Wang, Jiapeng Chen, Lujing Chen, Li Shi, Hongbing Liu

**Affiliations:** 1 Key Laboratory of Marine Drugs, Chinese Ministry of Education, School of Medicine and Pharmacy, Ocean University of China, Qingdao 266003, China; wangpengrui@stu.ouc.edu.cn (P.W.); cjp@stu.ouc.edu.cn (J.C.); clj3855@stu.ouc.edu.cn (L.C.); sl7613@stu.ouc.edu.cn (L.S.); 2 Laboratory for Marine Drugs and Bioproducts, Pilot National Laboratory for Marine Science and Technology (Qingdao), Qingdao 266237, China

**Keywords:** volatile organic compounds, seaweeds, characteristic VOCs, chemotaxonomy, biomedical utilization, headspace solid phase microextraction

## Abstract

Plant volatile organic compounds (VOCs) represent a relatively wide class of secondary metabolites. The VOC profiles of seven seaweeds (*Grateloupia filicina*, *Polysiphonia senticulosa*, *Callithamnion corymbosum*, *Sargassum thunbergii*, *Dictyota dichotoma*, *Enteromorpha prolifera* and *Ulva lactuca*) from the Yellow Sea of China were investigated using multifiber headspace solid phase microextraction coupled with gas chromatography–mass spectrometry (HS-SPME/GC–MS), among them, the VOCs of three red algae Grateloupia filicina, Polysiphonia senticulosa, and Callithamnion corymbosum were first reported. Principal component analysis (PCA) was used to disclose characteristic categories and molecules of VOCs and network pharmacology was performed to predict potential biomedical utilization of candidate seaweeds. Aldehyde was found to be the most abundant VOC category in the present study and (*E*)-*β*-ionone was the only compound found to exist in all seven seaweeds. The chemical diversity of aldehydes in *E. prolifera* suggest its potential application in chemotaxonomy and hinted that divinylbenzene/carboxen/polydimethylsiloxane (DVB/CAR/PDMS) fiber is more suitable for aldehyde extraction. VOCs in *D. dichotoma* were characterized as sesquiterpenes and diterpenes and the most relevant pharmacological pathway was the neuroactive ligand–receptor interaction pathway, which suggests that *D. dichotoma* may have certain preventive and therapeutic values in cancer, especially in lung cancer, in addition to neuropsychiatric diseases.

## 1. Introduction

Plant volatile organic compounds (VOCs) are produced by a range of physiological processes in many different plant tissues and typically occur as a complex mixture of lipophilic compounds with extremely diverse structures [1]. Their low molecular weight and high vapor pressure under ambient conditions allow them to freely exit through cellular membranes and reach the surrounding environment [2].

Plant VOCs have been reported in different geographical conditions ranging from Mediterranean environments [3] and tropical rainforests [4] to various extreme environments [5]. In aquatic ecosystems, algae are the main emitter of VOCs and the extraction of VOCs using headspace solid phase microextraction (HS-SPME) technology has been reported from brown algae [6], red algae [7] and green algae [8]. Algae release an abundance of VOCs to increase their tolerance to abiotic stresses, transfer stress information to homogeneous algae to induce defense, play allelopathic roles on heterogeneous algae and aquatic macrophytes for competing nutrients, or protect against predators [9]. In addition, the volatile contaminant in algae caught the interest of environmentalists [10]. Most of the studies on marine algae VOCs have focused on their allelopathy effects, whereas their contributions in other areas have been seldom investigated. VOCs are released under biotic or abiotic stresses and have great significance for plant survival and reproduction. In many cases, evolution has resulted in some plant VOCs acting as metabolic safety valves, protective or defense compounds, or communication cues [1]. VOCs can be divided chemically as aldehydes, alcohols, terpenoids, ketones, halogenated compounds, sulfur compounds and hydrocarbons and exhibit multitudinous functions, such as in being feeding attractants [11], pheromones [12] involved in chemical defenses [13,14,15] and acting as antimicrobial agents [16].

In the present study, VOC profiles of seven seaweeds blooming in the littoral area of Qingdao (the Yellow Sea of China) were investigated, including the first report of VOCs for three red algae species—*Grateloupia filicina*, *Polysiphonia senticulosa* and *Callithamnion corymbosum*. Characteristic categories and molecules of VOCs are discussed from the point of view of their chemotaxonomic significance and bioactivities are discussed in order to further expand these seaweeds’ potential utilities in pharmaceutical areas. Our study provides new insights for the chemotaxonomy of algae and for the biomedical application of *D. dichotoma.*

## 2. Results and Discussion

### 2.1. Headspace VOC Composition of Seaweeds

**Figure 1:**
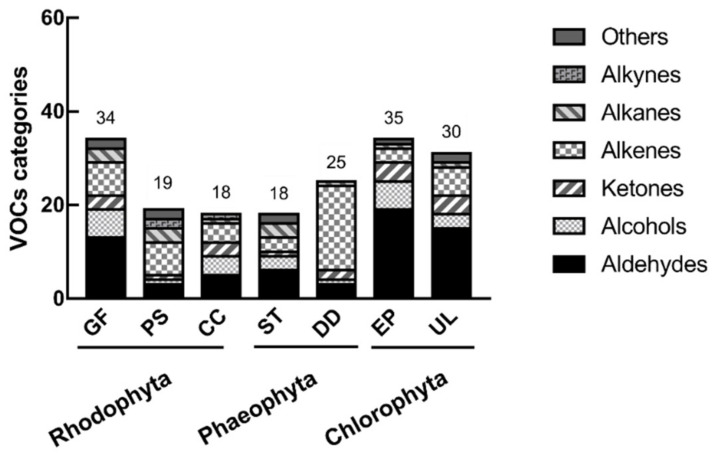
The number of volatile organic compound (VOC) categories identified in seaweeds GF—*Grateloupia filicina*, PS—*Polysiphonia senticulosa*, CC—*Callithamnion corymbosum*, ST—*Sargassum thunbergii*, DD—*Dictyota dichotoma*, EP—*Enteromorpha prolifera* and UL—*Ulva lactuca*.

VOC compositions were investigated in seven algae using headspace solid phase microextraction coupled with gas chromatography–mass spectrometry (HS-SPME/GC–MS) and are reported according to their phylum. The number of VOC categories identified in each alga are given in Figure 1.

#### 2.1.1. Headspace VOC Composition of Rhodophyta

There were 34, 19 and 18 compounds identified from *Grateloupia filicina*, *Polysiphonia senticulosa* and *Callithamnion corymbosum*, respectively (Table 1), and, among them, four compounds were common to all three species, including two norisoprenoids (*β*-cyclocitral and (*E*)-*β*-ionone), one alkene (3,5,5-trimethyl-1-hexene) and one alkane (heptadecane). This is the first report on the headspace VOC composition for these three red algae species.

The VOCs of *G. filicina* exhibited the most chemical diversity, with the most abundant being pentadecanal (20.11% in polydimethylsiloxane (PDMS) fiber), tridecanal (19.20% in PDMS fiber), 1-hexen-3-ol (19.58% in divinylbenzene/carboxen/polydimethylsiloxane (DVB/CAR/PDMS) fiber) and 1-octen-3-ol (18.72% in DVB/CAR/PDMS fiber). Wang Xiu-juan et al. [17] analyzed the semi-volatile organic compounds (SVOCs) of *G. filicina* using ethyl acetate as an extraction solvent and found that the main SVOCs were aldehydes, fatty acids and alcohols, with pentadecanal having the highest concentration. Our VOC analysis results are consistent with the results for SVOCs in the literature, suggesting that aldehydes play an important role in the metabolism of *G. filicina.*

Both *P. senticulosa* and *C. corymbosum* are from the same taxonomical order, Ceramiales Oltmanns and were found to contain two common compounds, 5-methyl-1-undecene and 1-undecyne, which were not found in *G. filicina*. Moreover, ectocarpene (21.83% in DVB/CAR/PDMS fiber; 14.71% in PDMS fiber) was a uniquely abundant compound in *P. senticulosa.*

#### 2.1.2. Headspace VOC Composition of Phaeophyta

The VOC compositions of brown algae *Sargassum thunbergia* and *Dictyota dichotoma* are reported in Table 2. Four compounds were commonly found, including one norisoprenoid ((*E*)-*β*-Ionone), one alkane (pentadecane) and two aldehydes (tridecanal and pentadecanal).

The most abundant VOCs in *S. thunbergia* are 8-heptadecene (5.97% in DVB/CAR/PDMS fiber; 14.82% in PDMS fiber), pentadecane (9.24% in DVB/CAR/PDMS fiber; 13.87% in PDMS fiber), pentadecanal (11.33% in PDMS fiber) and tridecanal (11.46% in DVB/CAR/PDMS fiber; 11.02% in PDMS fiber). A low relative content of 8-heptadecene (1.70%) has been reported in *S. thunbergia*, also using the SPME method though the fiber type was not mentioned [18]. Our data suggest that the choice of fiber type in the HS-SPME method has a decisive influence on the study’s conclusion. In addition, two types of volatile polyenes were reported to have been found in an essential oil prepared by a simultaneous distillation extraction method from *S. thunbergia* [19], although no polyenes were found in our study.

Fifteen sesquiterpenes were found in *D. dichotoma*, with the predominant one being germacrene D (34.83% in DVB/CAR/PDMS fiber; 62% in PDMS fiber) together with minor ones such as cadinene, muurolene and bourbonene. Compared with the VOC results (PDMS/DVB fiber) from *D. dichotoma* collected from the Adriatic Sea [20], we identified a lower number of sesquiterpenes. The reason for this difference may be that the profile of plant secondary metabolites may vary in different environments, or that different extract fibers have differing abilities to capture sesquiterpenes.

It has been reported that a total of 233 diterpenes were isolated from *Dictyota* species, most of which were from *D. dichotoma* [21]. The present study firstly reports three volatile diterpenes—1,5,9-trimethyl-12-(1-methylethyl)-4,8,13-cyclotetradecatriene-1,3-diol, thunbergol and geranyl-*α*-terpinene in *Dictyota*. 1,5,9-Trimethyl-12-(1-methylethyl)-4,8,13-cyclotetradecatriene-1,3-diol is a macrocyclic diterpene and is used as a representative flavor compound in tobacco [22]. Geranyl-*α*-terpinene is distributed in some Compositae plants, as well as existing in the volatiles of beech buds, which act as semiochemical attractants for the beech leaf-mining weevil, *Orchestes fagi* [23].

#### 2.1.3. Headspace VOC Composition of Chlorophyta

The results for two green algae, *Enteromorpha prolifera and Ulva lactuca*, are presented in Table 3. In total, 40 VOCs were identified and, among these, 21 were aldehydes.

The top three compounds with the highest content in *E. prolifera* were aldehydes, consisting of pentadecanal (8.49% in DVB/CAR/PDMS fiber; 24.4% in PDMS fiber), (*E*)-2-heptenal (9.64% in DVB/CAR/PDMS fiber) and tetradecanal (3.34% in DVB/CAR/PDMS fiber; 6.32% in PDMS fiber). It has been reported that significant differences were observed in the composition and types of VOCs in different harvest seasons for *E. prolifera* [24]. Differently from *E. prolifera*, the top three compounds with the highest content found in *U. lactuca* were not aldehydes, but alkenes and a ketone, including 8-heptadecene (5.32% in DVB/CAR/PDMS fiber; 19.70% in PDMS fiber), (*E*)-*β*-ionone (8.69% in DVB/CAR/PDMS fiber; 18.13% in PDMS fiber) and 3-ethyl-1,4-hexadiene (10.17% in DVB/CAR/PDMS fiber).

For *U. lactuca*, 30 VOCs were identified. Among them, 25 compounds also existed in *E. prolifera*, including 11 aldehydes ((*E*)-2-heptenal, 2,4-nonadienal, decanal, (*E*,*E*)-2,4-decadienal, undecanal, (*E*)-4,5-epoxydec-2-enal, tridecanal, tetradecanal, *Z*-11-pentadecenal, pentadecanal, (Z,Z,Z)-7,10,13-hexadecatrienal); four norisoprenoids (*β*-cyclocitral, *α*-ionone, (*E*)-geranylacetone, (*E*)-*β*-ionone); four alkenes (3,5-dimethyl-1-hexene, 3-ethyl-1,4-hexadiene, 1,2-dimethyl-cycloheptene, 8-heptadecene); two alkanes (1-butenylidene-cyclohexane, heptadecane); three alcohols ((*E*)-2-undecen-1-ol, 2,4-dimethyl-cyclohexanol, (*Z,Z*)-6,9-pentadecadien-1-ol); one ketone (6,10-dimethyl-2-undecanone) and one furan derivative (2-propyl-furan). The above common constituents in the two green algae covered almost all the VOC categories, differently from the red and brown algae.

(E)-β-Ionone was the only compound found to existing in all seven algae characterized in this study and it is widely distributed in the plant kingdom. As a C_13_ norisoprenoid, (E)-β-ionone can be produced by secondary metabolism of β-carotene and can also be produced by thermal degradation and photooxidation of carotenoids [25,26]. It has been reported that (E)-β-ionone has a wide range of biological activities, including having a strong anticancer effect [27].

### 2.2. Characteristic VOC Molecules and Potential Application in Chemotaxonomy

#### 2.2.1. PCA on Total VOCs Variables

**Figure 2:**
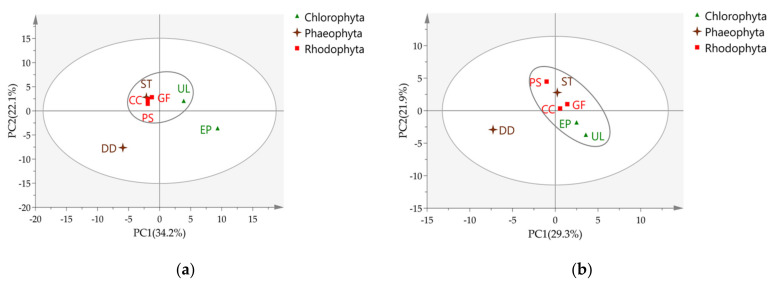
Principal component analysis (PCA) of VOC compositions in seven seaweeds determined by HS-SPME/GC–MS; score plot for (**a**) divinylbenzene/carboxen/polydimethylsiloxane (DVB/CAR/PDMS) fiber; (**b**) polydimethylsiloxane (PDMS) fiber.

Principal component analysis (PCA) on data from fiber DVB/CAR/PDMS exhibited that, except for *D. dichotoma* and *E. prolifera*, the other five seaweeds were clustered together (Figure 2a). The PCA results for fiber PDMS show that *D. dichotoma* was distinct from the other six seaweeds (Figure 2b).

*D. dichotoma* is an outlier in PCA, being very different from the other six seaweeds. The molecules that contributed most to this difference were revealed in the loading plot and are reported in the Supplementary Materials (Figure S1 and Table S1). As expected, the main differences were regarding sesquiterpenes and diterpenes.

Based on the revised biogenetic scheme that is widely cited, *Dictyota* diterpenes can be divided into three groups (I–III), resulting from the first formal cyclization of the geranylgeraniol precursor, which makes them potentially useful as chemotaxonomic and phylogenetic markers [28]. Unfortunately, the number of diterpenes available in *Dictyota* VOCs is very limited, making it difficult for them to be used in chemotaxonomy.

On the other hand, *Dictyota* contains large numbers of sesquiterpenes, distinguishing them from other seaweeds in this study. It is still not clear whether volatile sesquiterpenes can be used as chemotaxonomic markers for species identification of genus *Dictyota*, but it is worth exploring this through the VOC approach.

#### 2.2.2. PCA on Aldehyde Variables

**Figure 3:**
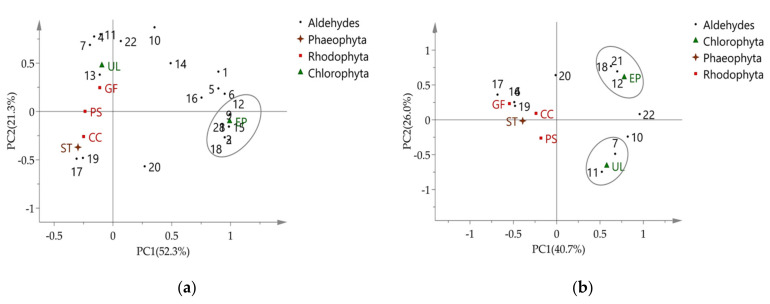
Bi-plot of PCA using aldehydes: (**a**) DVB/CAR/PDMS fiber; (**b**) PDMS fiber.

Using all the VOC data as PCA variables, *E. prolifera* was found to be an outlier, as shown in Figure 2a (DVB/CAR/PDMS fiber), but not in Figure 2b (PDMS fiber). Considering that aldehydes are the most abundant VOC categories (Figure 1), aldehydes were used as variables in PCA to reveal chemical differences between species. The results show that *E. prolifera* was separated well from other seaweeds (Figure 3). The aldehydes that contribute the most to species differences are shown in Table 4. There are six aldehydes characteristically distributed in *E. prolifera*.

Aldehydes are substances released by algae under biotic or abiotic stress and some aldehydes may induce the synthesis of a series of oxylipins in algae and therefore act as inducers of metabolic responses [29]. In view of the limited species and numbers of seaweeds in the present study, our data can only suggest that aldehydes may be of important value for the chemotaxonomic significance of *E. prolifera* and that the DVB/CAR/PDMS fiber is more suitable for headspace solid phase microextraction for aldehydes.

The volatile components of red algae *Bangia fuscopurpurea*, *Gelidium latifolium*, *Callithamnion granulatum*, *Ceramium elegans*, *Laurencia papillosa* and *Laurencia coronopus* from Black sea was detected and found that hydrocarbons can be used as chemotaxonomic markers of the two classes Bangiophyceae and Florideophyceae [30]. The chemotaxonomic significance was also discussed according to VOCs detection in two brown algae: *Taonia atomaria* and *Padina pavonica* [6]. The VOCs were used in evolutionary relationship discussion between the algae and liverwort *Fossombronia angulosa* due to the similarity [31].

### 2.3. Network Pharmacology and Potential Biomedical Application of D. Dichotoma

VOCs in *D. dichotoma* are characterized by sesquiterpenes and diterpenes. It has been reported that a total of 78 structurally diverse diterpenes have been isolated from *D. dichotoma* and exhibit multi-biological properties, such as cytotoxic, antitumor, antiviral, antifouling, antioxidant, antibacterial and antifungal activities [21]. In the present study, a network pharmacology method was performed to verify and discover new bioactivities of *D. dichotoma*.

**Figure 4:**
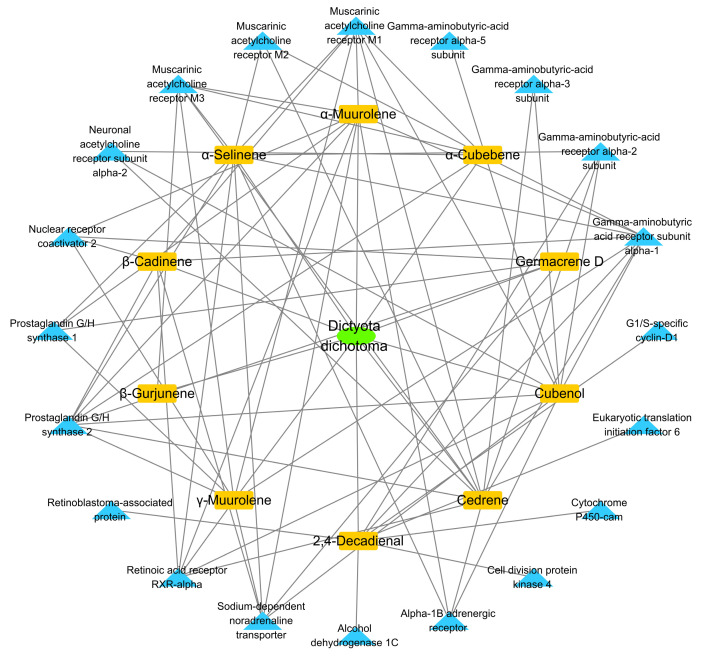
Compound–target interaction network of Dictyota dichotoma.

First, the VOCs of *D. dichotoma* were entered into the Traditional Chinese Medicine Systems Pharmacology Database and Analysis Platform (TCMSP) in the “chemical name” search box to obtain relevant targets. The “compound–target” relationships are shown in Figure 4, with 31 nodes (1 alga, 10 compounds and 20 targes). Comparing with the average degree score, 6.07, from network topology analysis, six key compounds were disclosed as cedrene, α-selinene, cubenol, *α*-muurolene, 2,4-decadienal and *γ*-muurolene, of which most are sesquiterpenes. The first three compounds are cedrene, α-selinene and cubenol, each of which can act on 10 targets.

**Figure 5:**
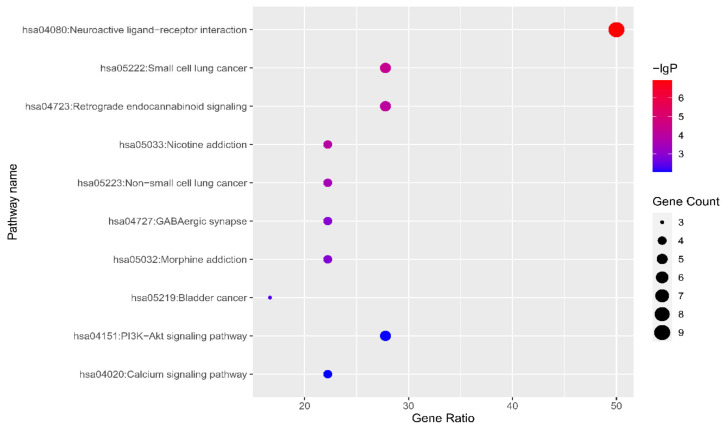
Bubble chart of pathway enrichment information.

Next, the Kyoto Encyclopedia of Genes and Genomes (KEGG) enrichment analysis was used to obtain the potential pathway in which VOCs might play a role. The 10 identified pathways (*p* < 0.01) are shown in Figure 5: neuroactive ligand–receptor interaction, small cell lung cancer, retrograde endocannabinoid signaling, nicotine addiction, non-small cell lung cancer, GABAergic synapse, morphine addiction, bladder cancer, PI3K–Akt signaling pathway and the calcium signaling pathway.

According to the literature, the neuroactive ligand–receptor interaction pathway plays an important role in lung cancer etiology [32]. Considering other pathways such as small cell lung cancer, non-small cell lung cancer, retrograde endocannabinoid signaling and PI3K–Akt signaling, we supposed that *D. dichotoma* may have certain preventive and therapeutic value in cancer, especially in lung cancer. The non-polar fractions of *D.dichotoma* exhibit anticancer activity in vitro, including the lung adenocarcinoma cell line (A-549) [33].

In addition, it should be noted that the neuroactive ligand–receptor interaction pathway is the most relevant pathway for VOCs in *D. dichotoma*, with nine target genes involved—CHRM1, CHRM2, CHRM3, CHRNA2, GABRA1, GABRA2, GABRA3, GABRA5 and ADRA1B. The neuroactive ligand–receptor interaction pathway is involved in environmental information processing as well as signaling molecules and interactions and an association has been found with certain neuropsychiatric disorders [34]. Therefore, *D. dichotoma* may have some potential use in the study of some neuropsychiatric diseases.

## 3. Materials and Methods

### 3.1. Sample Collection

The samples of seven algae were single-point collected from the coast of Qingdao city (120°20′30″ E, 36°3′43″ N, the Yellow Sea), Shandong Province, China, in July 2019. All of the studied algae are local common species, including *Grateloupia filicina* (Wulfen) C. Agardh, 1822 (Halymeniaceae Bory, Gigartinales Schmitz, Rhodophyceae), *Polysiphonia senticulosa* Harvey,1862 (Rhodomelaceae Areschoug, Ceramiales Oltmanns, Rhodophyceae), *Callithamnion corymbosum* (Smith) Lyngbye, 1819 (Ceramiaccae Dumoritier, Ceramiales Oltmanns, Rhodophyceae), *Sargassum thunbergii* (Mertens ex Roth) O’Kuntze, 1893 (Sargassaceae Kuetzing, Fucales Kylin, Phaeophyta), *Dictyota dichotoma* (Hudson) J. V. Lamouroux, 1809a (Dictyotaceae Lamourous ex Dumortier, Dictyotales Kjellman in Engler et Prantl, Phaeophyta), *Enteromorpha prolifera* (O.F.Müller) J. Agardh, 1883 (Ulvales Blackman et Tansley, Ulvaceae Lamourous ex Dumortier, Chlorophyta), *Ulva lactuca* Linneaeus, 1753 (Ulvales Blackman et Tansley, Ulvaceae Lamourous ex Dumortier, Chlorophyta). The voucher specimens have been deposited in the Key Laboratory of Marine Drugs, Chinese Ministry of Education, School of Medicine and Pharmacy, Ocean University of China.

The samples were separately collected and placed in an air-tight plastic bag containing surrounding seawater and were immediately transported to the laboratory. Before extraction, each sample were cut into small pieces and excess water was removed using filter paper.

### 3.2. Headspace Solid Phase Microextraction (HS-SPME)

HS-SPME was performed with a manual SPME holder. Four types of fibers obtained from Supelco Co. (Bellefonte, PA, USA) were compared for their adsorption properties in pilot experiments and two fibers, divinylbenzene/carboxen/polydimethylsiloxane (DVB/CAR/PDMS, 50/30 μm) and polydimethylsiloxane (PDMS, 100 μm) were finally, chosen. Fibers polyacrylate (PA, 85 μm) and 7 μm PDMS were not used in the present study. The fibers were activated in advance, according to the instructions.

A 1 g amount of prepared sample was placed into 4 mL glass vials and sealed using a cover with a Teflon gasket to provide a closed environment. The vials were placed at 60 °C for 15 min to equilibrate and the fiber was then pushed into the vial and maintained there for 20 min to adsorb the VOCs of the sample. After absorption, the fiber was removed from the vial and inserted into the injector (250 °C) of the GC–MS and kept for 6 min (DVB/CAR/PDMS fiber) or 4 min (PDMS fiber) to realize desorption. HS-SPME was performed in triplicate for each sample.

### 3.3. Gas Chromatography–Mass Spectrometry (GC–MS) Analyses

GC–MS analyses was performed on a Thermo Trace 1300 ISQ gas chromatography-mass spectrometer (Thermo Fisher Scientific Inc., San Jose, CA, USA) equipped with a TG-5MS capillary column (5% phenyl-methylpolysiloxane, 30 m × 0.25 mm, 0.25 μm, Thermo Fisher Scientific Inc.). For DVB/CAR/PDMS fiber, the oven temperature was initially set at 70 °C and maintained for 1 min, increased at a rate of 3 °C/min to 100 °C and continually increased at a rate of 5 °C/min to 200 °C, followed by a final hold at this temperature for 2 min. For PDMS fiber, the oven temperature was initially maintained at 70 °C for 2 min, increased from 70 to 200 °C at 3 °C/min and then held at 200 °C for 6 min. Helium (99.9% purity) was used as the carrier gas at a flow rate of 1 mL/min. The temperature of the injector, detector transfer line and ion source were 250, 270 and 230 °C, respectively. The MS detector was operated in the full scan mode and the electron energy was 70 eV and the scan range was from *m/z* 30 to 350 amu. All analyses were performed in triplicate.

Visualization, calibration and normalization of the GC–MS data were performed using Xcalibur 2.1 (Thermo Fisher Scientific Inc.). The VOC peaks were identified by comparison of their retention index (RI) and MS data with data in the NIST Chemistry WebBook [35] and NIST 11 MS Data Library, respectively. The RI values of the VOCs were calculated by analyzing the C7–C30 *n*-alkanes (Sigma-Aldrich, St. Louis, MO, USA) under the same GC–MS conditions as samples. Authentic standards included trans-2-nonenal (Shanghai Macklin Biochemical Co., Ltd., Shanghai, China), tridecanal (Shanghai Macklin Biochemical Co., Ltd.), 1-octen-3-ol (Shanghai Macklin Biochemical Co., Ltd.), tetradecanal (Shanghai Aladdin Biochemical Technology Co., Ltd., Shanghai, China), germacrene D (Toronto Research Chemicals, Toronto, ON, Canada) and (*E*)-*β*-ionone (Xiya Chemical Technology Co., Ltd., Chengdu, China), which were used for confirming VOC identity. All VOC peaks were quantified by area normalization with consistent peak parameters (baseline, area noise and peak noise) prior to data analysis and statistics.

### 3.4. Chemometrics and Network Pharmacology Analysis

The principal components analysis (PCA) of all VOCs was conducted using SIMCA 14.1. The targets of identified volatile components were obtained from the Traditional Chinese Medicine Systems Pharmacology Database and Analysis Platform (TCMSP, https://tcmspw.com/tcmsp.php, accessed on 21 December 2020) [36], the gene information of the related targets was taken from the Uniport database (https://www.Unitprot.org/, accessed on 21 December 2020) and the String database (https://string-db.org/, accessed on 21 December 2020) [37,38] and KEGG pathway enrichment analysis were accessed through DAVID software (https://david.ncifcrf.gon/, accessed on 21 December 2020) [39] with the corresponding bubble chart realized using the R program. The interactive component–target relationships in algae were visualized using Cytoscape 3.7.2 software.

## 4. Conclusions

The volatile composition of seven algae from Yellow Sea of China was detected by HS-SPME/GC-MS, the VOCs of three red algae *Grateloupia filicina*, *Polysiphonia senticulosa* and *Callithamnion corymbosum* among them are first reported. The PCA analysis of VOCs reveals the chemotaxonomy significance of aldehydes in green algae *Enteromorpha prolifera* and sesquiterpenes in brown algae *Dictyota dichotoma*, as well as the applicability of DVB/CAR/PDMS fiber in volatile aldehydes. The network pharmacology analysis of *Dictyota dichotoma* indicate the potential biomedical application in fields of lung cancer and neuropsychiatric diseases. Our study exploits the new research value of VOCs in algae from the perspective of chemotaxonomy and network pharmacology.

## Figures and Tables

**Table 1:** VOC composition in Rhodophyta, determined by headspace solid phase microextraction coupled with gas chromatography–mass spectrometry (HS-SPME/GC–MS).

NO.	Compound	Molecular Formula	Compound Class	RI ^a^	Area Percentage (%)			Identification
					GF ^b^	PS ^b^	CC ^b^	
**1**	(*E,E,E*)-2,4,6-Octatriene ^T^	C_8_H_12_	Alkene	<800	1.03 *	0.00	0.00	MS
**2**	3,5,5-Trimethyl-1-hexene ^T^	C_9_H_18_	Alkene	<800	8.24 *	1.74 *	2.74 *	MS
**3**	3,5-Dimethyl-1-hexene ^T^	C_8_H_16_	Alkene	<800	0.36 *	0.00	0.00	MS
**4**	1-Hexen-3-ol ^T^	C_6_H_12_O	Alcohol	<800	19.58 *	0.00	4.44 *	MS
**5**	2-Propyl-furan ^T^	C_7_H_10_O	Furan derivative	<800	0.30 *	1.02 *	0.00	MS
**6**	3-Ethyl-1,4-hexadiene ^T^	C_8_H_14_	Alkene	846	1.16 *	1.25 *	0.00	MS
**7**	2-Methylpropylidene-Cyclopentane	C_9_H_16_	Alkene	908	1.48 *	0.00	0.00	MS, RI
**8**	(*Z*)-2-Octen-1-ol ^T^	C_8_H_16_O	Unsaturated alcohol	927	8.5 ^#^	0.00	0.00	MS
**9**	Isocumene	C_9_H_12_	Others	931	0.00	1.67 *	0.00	MS, RI
**10**	3-Cyclohexene-1-ethanol	C_8_H_14_O	Alcohol	935	2.90 ^#^	0.00	0.00	MS.RI
**11**	(*E*)-2-Heptenal	C_7_H_12_O	Aldehyde	960	4.78 *	1.51 *	0.00	MS, RI
**12**	1-Octen-3-ol ^S^	C_8_H_16_O	Alcohol	980	18.72 *	0.00	2.15 *	MS, RI
**13**	3,7-Dimethyl-1-octene ^T^	C_10_H_20_	Alkene	985	0.00	0.00	7.81 *	MS
**14**	2,7-Octadien-1-ol ^T^	C_8_H_14_O	Unsaturated alcohol	986	8.39 *	0.00	0.00	MS
**15**	4-Methyl-2-propyl-1-pentanol ^T^	C_9_H_20_O	Alcohol	990	0.00	0.00	3.59 ^#^	MS
**16**	5-Methyl-1-undecene ^T^	C_12_H_24_	Alkene	1025	0.00	6.91 *	12.70 *	MS
**17**	(*E*)-2-Undecen-1-ol ^T^	C_11_H_22_O	Unsaturated alcohol	1039	0.25 *	1.28 *	0.00	MS
**18**	*(9Z)*-1,9-Dodecadiene ^T^	C_12_H_22_	Alkene	1092	0.00	0.00	1.77 ^#^	MS
**19**	Ectocarpene ^T^	C_11_H_16_	Alkene	1105	0.00	21.83 *, 14.71 ^#^	0.00	MS
**20**	1-Undecyne	C_11_H_20_	Alkyne	1108	0.00	2.46 *	8.86 *	MS, RI
**21**	Dictyopterene D’ ^T^	C_11_H_18_	Alkene	1112	0.00	1.74 *, 0.95 ^#^	0.00	MS
**22**	(*Z*)-6-Nonenal	C_9_H_16_O	Aldehyde	1113	3.25 *	0.00	0.00	MS, RI
**23**	Decanal	C_10_H_20_O	Aldehyde	1173	0.07 *	0.00	0.00	MS, RI
**24**	β-Cyclocitral	C_10_H_16_O	C_10_-Norisoprenoid	1192	0.79 *, 0.69 ^#^	1.55 *	0.95 *	MS, RI
**25**	β-Cyclohomocitral	C_11_H_18_O	Aldehyde	1236	0.07 *	0.00	0.00	MS, RI
**26**	2,4-Decadienal	C_10_H_16_O	Unsaturated aldehyde	1275	0.33 *	0.00	0.00	MS, RI
**27**	Undecanal	C_11_H_22_O	Aldehyde	1290	0.47 *, 0.52 ^#^	0.00	0.00	MS, RI
**28**	(*E*,*E*)-2,4-Decadienal	C_10_H_16_O	Unsaturated aldehyde	1298	0.98 *, 0.66 ^#^	0.69 *	0.00	MS, RI
**29**	Dodecanal	C_12_H_24_O	Aldehyde	1400	0.37 *, 0.95 ^#^	0.00	0.00	MS, RI
**30**	α-Ionone	C_13_H_20_O	C_13_-Norisoprenoid	1420	1.50 *, 2.01 ^#^	0.00	1.16 *, 1.60 ^#^	MS, RI
**31**	Dihydropseudoionone	C_13_H_22_O	C_13_-Norisoprenoid	1447	0.15 *, 0.40 ^#^	0.00	0.00	MS, RI
**32**	(*E*)-β-Ionone ^S^	C_13_H_20_O	C_13_-Norisoprenoid	1481	2.65 *, 5.68 ^#^	1.37 *	2.39 *, 4.01 ^#^	MS, RI
**33**	1-Pentadecene	C_15_H_30_	Alkene	1484	3.84 *, 2.33 ^#^	1.33 ^#^	0.00	MS, RI
**34**	Pentadecane ^S^	C_15_H_32_	Alkane	1492	0.57 *, 0.88 ^#^	1.39 *, 11.19^#^	0.00	MS.RI
**35**	Tridecanal ^S^	C_13_H_26_O	Aldehyde	1503	5.55 *, 19.20 ^#^	0.00	2.28 *, 5.60 ^#^	MS, RI
**36**	Cyclopentadecane ^T^	C_15_H_30_	Alkane	1508	0.31 *	1.16 ^#^	0.00	MS
**37**	Dihydroactinidiolide	C_11_H_16_O_2_	C_11_-Norisoprenoid	1525	0.06 *	0.00	0.00	MS, RI
**38**	Tetradecanal**^S^**	C_14_H_28_O	Aldehyde	1608	1.75 ^#^	0.00	1.16 ^#^	MS, RI
**39**	8-Heptadecene	C_17_H_34_	Alkene	1681	0.10 *	2.85^#^	0.00	MS, RI
**40**	*Z*-11-Pentadecenal ^T^	C_15_H_28_O	Aldehyde	1689	0.26 *, 1.78 ^#^	0.00	1.09 ^#^	MS
**41**	Heptadecane ^S^	C_17_H_36_	Alkane	1693	1.73 *, 5.76 ^#^	3.19 *, 15.93 ^#^	2.04 *, 3.15 ^#^	MS, RI
**42**	Pentadecanal	C_15_H_30_O	Aldehyde	1711	3.23 *, 20.11 ^#^	0.00	11.64 *, 35.43 ^#^	MS, RI
**43**	Perhydrofarnesyl acetone	C_18_H_36_O	Ketone	1840	0.00	0.00	0.91 *, 3.14 ^#^	MS, RI
**44**	1,11-Dodecadiyne ^T^	C_12_H_18_	Alkyne	1845	0.00	0.81 ^#^	0.00	MS
**45**	(*Z,Z*)-6,9-Pentadecadien-1-ol ^T^	C_15_H_28_O	Unsaturated alcohol	1889	0.00	0.00	0.46 *, 1.92 ^#^	MS
Total Identified (%)					90.59 *, 74.10 ^#^	49.61 *, 48.92 ^#^	60.53 *,62.46 ^#^	

^T^ tentatively identified. ^S^ identified by mass spectra (MS) and retention index (RI) compared with authentic standard. ^a^ RI: retention index relative to C7-C30 alkanes. ^b^ GF—*Grateloupia filicina*; PS—*Polysiphonia senticulosa*; CC—*Callithamnion corymbosum*. * data from fiber DVB/CAR/PDMS. ^#^ data from fiber PDMS.

**Table 2:** VOC composition in brown algae, determined by headspace solid phase microextraction coupled with gas chromatography–mass spectrometry (HS-SPME/GC–MS).

NO.	Compound	Molecular Formula	Compound Class	RI ^a^	Area Percentage (%)		Identification
					ST ^b^	DD ^b^	
**1**	2-Propyl-furan ^T^	C_7_H_10_O	Furan derivative	<800	1.37 *	0.00	MS
**2**	(*E*)-2-Hepten-1-ol ^T^	C_7_H_14_O	Alcohol	830	0.00	4.62 *	MS
**3**	Sulcatone ^T^	C_8_H_14_O	C_13_-Norisoprenoid	852	0.00	5.21 *	MS
**4**	2-Propyl-1-pentanol ^T^	C_8_H_18_O	Alcohol	896	0.82 *	0.00	MS
**5**	Isocumene	C_9_H_12_	Others	931	1.14 *	0.00	MS, RI
**6**	2,7-Dimethyl-1-octanol ^T^	C_10_H_22_O	Alcohol	1023	0.94 *	0.00	MS
**7**	(*E*)-2-Undecen-1-ol ^T^	C_11_H_22_O	Alcohol	1039	1.48 *	0.00	MS
**8**	Ectocarpene ^T^	C_11_H_16_	Alkene	1105	3.40 *	0.00	MS
**9**	β-Cyclocitral	C_10_H_16_O	C_10_-Norisoprenoid	1192	1.05 *	0.00	MS, RI
**10**	2,4-Decadienal	C_10_H_16_O	Unsaturated aldehyde	1275	0.00	0.72 *	MS, RI
**11**	α-Cubebene	C_15_H_24_	Sesquiterpene	1338	0.00	0.81 *	MS, RI
**12**	β-Bourbonene	C_15_H_24_	Sesquiterpene	1376	0.00	1.07 *, 1.39 ^#^	MS, RI
**13**	Cedrene	C_15_H_24_	Sesquiterpene	1418	0.00	0.28 ^#^	MS, RI
**14**	β-Copaene	C_15_H_24_	Sesquiterpene	1423	0.00	0.57 *	MS, RI
**15**	cis-Muurola-3,5-diene	C_15_H_24_	Sesquiterpene	1441	0.00	0.48 *	MS, RI
**16**	β-Gurjunene	C_15_H_24_	Sesquiterpene	1458	0.00	1.72 *	MS, RI
**17**	γ-Muurolene	C_15_H_24_	Sesquiterpene	1468	0.00	2.82 *, 0.62 ^#^	MS, RI
**18**	Germacrene D ^S^	C_15_H_24_	Sesquiterpene	1477	0.00	34.83 *, 62.00 ^#^	MS, RI
**19**	(*E*)-β-Ionone ^S^	C_13_H_20_O	C_13_-Norisoprenoid	1481	1.22 *, 0.93 ^#^	0.52 *, 0.44 ^#^	MS, RI
**20**	1-Pentadecene	C_15_H_30_	Alkene	1484	4.31 *, 5.46 ^#^	0.00	MS, RI
**21**	α-Selinene	C_15_H_24_	Sesquiterpene	1488	0.00	0.38 ^#^	MS, RI
**22**	Pentadecane ^S^	C_15_H_32_	Alkane	1492	9.24 *, 13.87 ^#^	5.94 *, 7.98 ^#^	MS.RI
**23**	Tridecanal ^S^	C_13_H_26_O	Aldehyde	1503	11.46 *, 11.02 ^#^	2.51 *, 1.74 ^#^	MS, RI
**24**	Cyclopentadecane ^T^	C_15_H_30_	Alkane	1508	2.94 *, 1.51 ^#^	0.00	MS
**25**	Muurola-4,9-diene	C_15_H_24_	Sesquiterpene	1510	0.00	3.37 *	MS, RI
**26**	β-Cadinene	C_15_H_24_	Sesquiterpene	1518	0.00	0.52 ^#^	MS, RI
**27**	δ-Cadinene	C_15_H_24_	Sesquiterpene	1520	0.00	5.28 *	MS, RI
**28**	1,4-Cadinadiene	C_15_H_24_	Sesquiterpene	1529	0.00	0.44 *	MS, RI
**29**	α-Muurolene ^T^	C_15_H_24_	Sesquiterpene	1535	0.00	0.82 *	MS
**30**	Tetradecanal ^S^	C_14_H_28_O	Aldehyde	1608	0.62 *, 0.48 ^#^	0.00	MS, RI
**31**	Cubenol	C_15_H_26_O	Sesquiterpene	1637	0.00	3.69 *, 4.70 ^#^	MS, RI
**32**	8-Heptadecene	C_17_H_34_	Alkene	1681	5.97 *, 14.82 ^#^	0.00	MS, RI
**33**	Heptadecane ^S^	C_17_H_36_	Alkane	1693	1.63 *, 1.59 ^#^	0.00	MS, RI
**34**	(*Z*)-11-Pentadecenal ^T^	C_15_H_28_O	Aldehyde	1689	5.49 *, 5.85 ^#^	0.00	MS
**35**	Pentadecanal	C_15_H_30_O	Aldehyde	1711	6.86 *, 11.33 ^#^	0.46 ^#^	MS, RI
**36**	(Z,Z,Z)-7,10,13-Hexadecatrienal ^T^	C_16_H_26_O	Unsaturated aldehyde	1890	0.91 ^#^	0.00	MS
**37**	1,5,9-Trimethyl-12-(1-methylethyl)-4,8,13-Cyclotetradecatriene-1,3-diol ^T^	C_20_H_34_O_2_	Diterpene	1989	0.00	5.70 ^#^	MS
**38**	Thunbergol	C_20_H_34_O_2_	Diterpene	2089	0.00	3.30 ^#^	MS, RI
**39**	Geranyl-α-terpinene ^T^	C_20_H_32_	Diterpene	>2100	0.00	1.04 *, 0.99 ^#^	MS
Total Identified (%)					59.94 *, 67.77 ^#^	76.46 *, 90.51 ^#^	

^T^ tentatively identified. ^S^ identified by mass spectra (MS) and retention index (RI) compared with authentic standard. ^a^ RI: retention index relative to C7–C30 alkanes. ^b^ ST—Sargassum thunbergii; DD—Dictyota dichotoma. * data from fiber DVB/CAR/PDMS. ^#^ data from fiber PDMS.

**Table 3:** VOC composition in green algae determined by headspace solid phase microextraction coupled with gas chromatography–mass spectrometry (HS-SPME/GC–MS).

NO.	Compound	Molecular Formula	Compound Class	RI ^a^	Area Percentage (%)		Identification
					EP ^b^	UL ^b^	
**1**	3,5,5-Trimethyl-1-hexene ^T^	C_9_H_18_	Alkene	<800	0.00	1.34 *	MS
**2**	3,5-Dimethyl-1-hexene ^T^	C_8_H_16_	Alkene	<800	3.75 *	1.27 *	MS
**3**	2-Propyl-furan ^T^	C_7_H_10_O	Furan derivatives	<800	4.61 *	4.74 *, 3.82 ^#^	MS
**4**	2,4-Octadiene	C_8_H_14_	Alkene	805	0.00	3.06 *	MS, RI
**5**	3-Ethyl-1,4-hexadiene ^T^	C_8_H_14_	Alkene	846	3.59 *	10.17 *	MS
**6**	(*E*)-2-Heptenal	C_7_H_12_O	Aldehyde	960	9.64 *	4.60 *	MS, RI
**7**	1,2-Dimethyl-cycloheptene ^T^	C_9_H_16_	Alkene	988	3.32 *	3.40 *	MS
**8**	4-Heptenal ^T^	C_7_H_12_O	Aldehyde	1001	2.94 *	0.00	MS
**9**	(4*E*)-2-Methyl-4-hexen-3-ol ^T^	C_7_H_14_O	Alcohol	1025	3.08 *	0.00	MS
**10**	(*E*)-2-Undecen-1-ol ^T^	C_11_H_22_O	Unsaturated alcohol	1039	3.15 *	1.27 *	MS
**11**	2,4-Dimethyl-Cyclohexanol	C_8_H_16_O	Alcohol	1045	0.80 *	1.90 *	MS, RI
**12**	3-Cyclohexene-1-carboxaldehyde ^T^	C_8_H_12_O	Aldehyde	1056	0.50 *	0.00	MS
**13**	(*Z*)-6-Nonenal	C_9_H_16_O	Aldehyde	1113	0.00	1.94 *	MS, RI
**14**	2,4-Nonadienal	C_9_H_14_O	Unsaturated aldehyde	1180	0.95 *	0.52 *	MS, RI
**15**	Decanal	C_10_H_20_O	Aldehyde	1173	1.36 *	0.58 *	MS, RI
**16**	9-Oxabicyclo [6.1.0]nonan-4-ol ^T^	C_8_H_14_O_2_	Alcohol	1186	0.70 *	0.00	MS
**17**	β-Cyclocitral	C_10_H_16_O	C_10_-Norisoprenoid	1192	0.89 *, 1.05 ^#^	3.05 *, 3.17 ^#^	MS, RI
**18**	(*Z*)-2-Decenal	C_10_H_18_O	Aldehyde	1243	0.70 *	0.00	MS, RI
**19**	Citral	C_10_H_16_O	C_10_-Norisoprenoid	1253	0.32 *	0.00	MS, RI
**20**	1-Butenylidene-cyclohexane ^T^	C_10_H_16_	Alkane	1260	0.81 *	1.80 *, 1.80 ^#^	MS
**21**	Undecanal	C_11_H_22_O	Aldehyde	1290	0.31 *	0.15 *	MS, RI
**22**	2,4-Decadienal	C_10_H_16_O	Unsaturated aldehyde	1275	0.00	0.96 *, 0.48 ^#^	MS, RI
**23**	(*E*,*E*)-2,4-Decadienal	C_10_H_16_O	Unsaturated aldehyde	1298	0.77 *, 1.61 ^#^	3.02 *, 2.59 ^#^	MS, RI
**24**	4,4,6-Trimethyl-cyclohex-2-en-1-ol	C_9_H_16_O	Alcohol	1330	0.28 *	0.00	MS, RI
**25**	2-Undecenal	C_11_H_20_O	Aldehyde	1353	0.65 *	0.00	MS, RI
**26**	(*E*)-4,5-Epoxydec-2-enal	C_10_H_16_O_2_	Unsaturated Aldehyde	1368	1.70 *, 0.85 ^#^	0.28 *	MS, RI
**27**	6,10-Dimethyl-2-undecanone	C_13_H_26_O	Ketone	1395	0.56 *	0.19 *	MS, RI
**28**	Dodecanal	C_12_H_24_O	Aldehyde	1400	0.43 *	0.00	MS, RI
**29**	α-Ionone	C_13_H_20_O	C_13_-Norisoprenoid	1420	0.21 *, 0.45 ^#^	2.59 *, 4.24 ^#^	MS, RI
**30**	(*E*)-Geranylacetone	C_13_H_22_O	C_13_-Norisoprenoid	1447	0.93 *, 1.74 ^#^	0.29 *, 0.48 ^#^	MS, RI
**31**	(*E*)-*β*-Ionone ^S^	C_13_H_20_O	C_13_-Norisoprenoid	1481	3.53 *, 5.97 ^#^	8.69 *, 18.13 ^#^	MS, RI
**32**	Tridecanal ^S^	C_13_H_26_O	Aldehyde	1503	1.31 *, 2.37 ^#^	0.37 *, 0.65 ^#^	MS, RI
**33**	Dihydroactinidiolide	C_11_H_16_O_2_	C_11_-Norisoprenoid	1525	0.00	0.29 *	MS, RI
**34**	Tetradecanal ^S^	C_14_H_28_O	Aldehyde	1608	3.34 *, 6.32 ^#^	0.79 ^#^	MS, RI
**35**	8-Heptadecene	C_17_H_34_	Alkene	1681	2.72 ^#^	5.32 *, 19.70 ^#^	MS, RI
**36**	*(Z)*-11-Pentadecenal ^T^	C_15_H_28_O	Aldehyde	1689	0.40 *, 0.81 ^#^	0.77 *	MS
**37**	Pentadecanal	C_15_H_30_O	Aldehyde	1711	8.49 *, 24.40 ^#^	3.83 *, 7.36 ^#^	MS, RI
**38**	*(Z)*-11-Hexadecenal	C_16_H_30_O	Aldehyde	1793	0.59 *, 1.18 ^#^	0.00	MS, RI
**39**	(*Z,Z*)-6,9-Pentadecadien-1-ol ^T^	C_15_H_28_O	Unsaturated alcohol	1889	0.83 ^#^	0.54 *, 0.93 ^#^	MS
**40**	(Z,Z,Z)-7,10,13-Hexadecatrienal ^T^	C_16_H_26_O	Unsaturated aldehyde	1890	0.46 *, 6.13 ^#^	2.83 *, 4.96 ^#^	MS
Total Identified (%)					65.07 *, 56.43 ^#^	69.79 *, 69.10 ^#^	

^T^ tentatively identified. ^S^ identified by mass spectra (MS) and retention index (RI) compared with authentic standard. ^a^ RI: retention index relative to C7–C30 alkanes. ^b^ EP—Enteromorpha prolifera; UL—Ulva lactuca. * data from fiber DVB/CAR/PDMS. ^#^ data from fiber PDMS.

**Table 4:** Various aldehydes in PCA loading plot, as shown in Figure 3.

No.	Aldehydes	Molecular Formula	Related Fiber	Algae
**2**	4-Heptenal	C_7_H_12_O	DVB/CAR/PDMS	EP
**3**	3-Cyclohexene-1-carboxaldehyde	C_8_H_12_O	DVB/CAR/PDMS	EP
**7**	*β*-Cyclocitral	C_10_H_16_O	PDMS	EP, UL ^Δ^, GF, PS, CC, ST
**8**	(*Z*)-2-Decenal	C_10_H_18_O	DVB/CAR/PDMS	EP
**9**	Citral	C_10_H_16_O	DVB/CAR/PDMS	EP
**11**	2,4-Decadienal	C_10_H_16_O	PDMS	UL ^Δ^, GF, DD
**12**	(*E*)-4,5-Epoxydec-2-enal	C_10_H_16_O_2_	DVB/CAR/PDMS, PDMS	EP ^Δ^, UL
**15**	2-Undecenal	C_11_H_20_O	DVB/CAR/PDMS	EP
**18**	Tetradecanal	C_14_H_28_O	DVB/CAR/PDMS, PDMS	EP ^Δ^, UL, GF, CC, ST
**21**	(*Z*)-11-Hexadecenal	C_16_H_30_O	DVB/CAR/PDMS, PDMS	EP

^Δ^ with the highest content.

## Data Availability

The data presented in this study are available in the manuscript.

## Supplementary Materials

The following are available online at https://www.mdpi.com/article/10.3390/md19040192/s1, Figure S1: PCA of VOC compositions in seven seaweeds determined by HS-SPME/GC–MS, loading plot with: (a) DVB/CAR/PDMS fiber; (b) PDMS fiber. Table S1: The compounds corresponding to dots with coordinates in the loading plot.

## References

[1] Penuelas J., Llusia J. (2004). Plant VOC emissions: Making use of the unavoidable. Trends Ecol. Evol..

[2] Pichersky E., Noel J.P., Dudareva N. (2006). Biosynthesis of plant volatiles: Nature’s diversity and ingenuity. Science.

[3] Owen S.M., Boissard C., Hewitt C.N. (2001). Volatile organic compounds (VOCs) emitted from 40 Mediterranean plant species: VOC speciation and extrapolation to habitat scale. Atmos. Environ..

[4] Kesselmeier J., Guenther A., Hoffmann T., Piedade M.T., Warnke J. (2009). Natural volatile organic compound emissions from plants and their roles in oxidant balance and particle formation. Amazonia and Global Change.

[5] Rinnan R., Steinke M., McGenity T., Loreto F. (2014). Plant volatiles in extreme terrestrial and marine environments. Plant Cell Environ..

[6] Jerkovic I., Kranjac M., Marijanovic Z., Roje M., Jokic S. (2019). Chemical diversity of headspace and volatile oil composition of two brown algae (*Taonia atomaria* and *Padina pavonica*) from the Adriatic Sea. Molecules.

[7] De Alencar D.B., Diniz J.C., Rocha S.A.S., Dos Santos Pires-Cavalcante K.M., Freitas J.O., Nagano C.S., Sampaio A.H., Saker-Sampaio S. (2017). Chemical composition of volatile compounds in two red seaweeds, *Pterocladiella capillacea* and *Osmundaria obtusiloba*, using static headspace gas chromatography mass spectrometry. J. Appl. Phycol..

[8] Yamamoto M., Baldermann S., Yoshikawa K., Fujita A., Mase N., Watanabe N. (2014). Determination of volatile compounds in four commercial samples of Japanese green algae using solid phase microextraction gas chromatography mass spectrometry. Sci. World J..

[9] Zuo Z. (2019). Why algae release volatile organic compounds—The emission and roles. Front. Microbiol..

[10] Rocha F., Homem V., Castro-Jimenez J., Ratola N. (2019). Marine vegetation analysis for the determination of volatile methylsiloxanes in coastal areas. Sci. Total Environ..

[11] Akakabe Y., Kajiwara T. (2008). Bioactive volatile compounds from marine algae: Feeding attractants. J. Appl. Phycol..

[12] Pohnert G., Boland W. (2002). The oxylipin chemistry of attraction and defense in brown algae and diatoms. Nat. Prod. Rep..

[13] Van Alstyne K.L., Houser L.T. (2003). Dimethylsulfide release during macroinvertebrate grazing and its role as an activated chemical defense. Mar. Ecol. Prog. Ser..

[14] Schnitzler I., Pohnert G., Hay M., Boland W. (2001). Chemical defense of brown algae (Dictyopteris spp.) against the herbivorous amphipod *Ampithoe longimana*. Oecologia.

[15] Wiesemeier T., Hay M., Pohnert G. (2007). The potential role of wound-activated volatile release in the chemical defence of the brown alga *Dictyota dichotoma*: Blend recognition by marine herbivores. Aquat. Sci..

[16] Kajiwara T., Matsui K., Akakabe Y., Murakawa T., Arai C. (2006). Antimicrobial browning-inhibitory effect of flavor compounds in seaweeds. J. Appl. Phycol..

[17] Wang X.J., Xu J.L., Yan X.J. (2010). Analysis of the semivolatile organic compounds of two seaweeds. Haiyang Kexue.

[18] Zhang M., Li R.X., Hu C.M., Yang L.E., Tang J., Lu Q.Q., Zhang T., Shen Z.G., Shen S.D., Xu P. (2014). The metabolism of 8-heptadecene in *Pyropia* (Bangiaceae, Rhodophyta). J. Appl. Phycol..

[19] Lu S.J., Yosemoto S., Satomi D., Handa H., Akakabe Y. (2018). Two types of volatile polyenes in the brown alga *Sargassum thunbergii*. J. Oleo Sci..

[20] Jerkovic I., Marijanovic Z., Roje M., Kus P.M., Jokic S., Coz-Rakovac R. (2018). Phytochemical study of the headspace volatile organic compounds of fresh algae and seagrass from the Adriatic Sea (single point collection). PLoS ONE.

[21] Chen J.Y., Li H., Zhao Z.S., Xia X., Li B., Zhang J.R., Yan X.J. (2018). Diterpenes from the marine algae of the genus *Dictyota*. Mar. Drugs.

[22] Roberts D.L., Rowland R.L. (1962). Macrocyclic diterpenes. α- and β-4,8,13-Duvatriene-1,3-diols from Tobacco. J. Org. Chem..

[23] Silk P.J., Mayo P.D., LeClair G., Brophy M., Pawlowski S., MacKay C., Hillier N.K., Hughes C., Sweeney J.D. (2017). Semiochemical attractants for the beech leaf-mining weevil, *Orchestes fagi*. Entomol. Exp. Appl..

[24] Miao F.F., Ding Y., Lin J.L., He H.P., Zhu W.C., Su X.R. (2014). Analysis of volatile compounds in *Enteromorpha prolifera* harvested during different seasons by electronic nose and HS-SPME-GC-MS. Mod. Food Sci. Technol..

[25] Reese K.L., Fisher C.L., Lane P.D., Jaryenneh J.D., Moorman M.W., Jones A.D., Frank M., Lane T.W. (2019). Chemical profiling of volatile organic compounds in the headspace of algal cultures as early biomarkers of algal pond crashes. Sci. Rep..

[26] Zhang K.J., Lin T.F., Zhang T.Q., Li C., Gao N.Y. (2013). Characterization of typical taste and odor compounds formed by *Microcystis aeruginosa*. J. Environ. Sci..

[27] Jones S., Fernandes N.V., Yeganehjoo H., Katuru R., Qu H.B., Yu Z.L., Mo H.B. (2013). *β*-Ionone induces cell cycle arrest and apoptosis in human prostate tumor cells. Nutr. Cancer.

[28] Teixeira V.L., Kelecom A. (1988). A chemotaxonomic study of diterpenes from marine brown algae of the genus *Dictyota*. Sci. Total Environ..

[29] Goulitquer S., Ritter A., Thomas F., Ferec C., Salauen J.P., Potin P. (2009). Release of volatile aldehydes by the brown algal kelp *Laminaria digitata* in response to both biotic and abiotic stress. ChemBioChem.

[30] Kamenarska Z., Ivanova A., Stancheva R., Stoyneva M., Stefanov K., Dimitrova-Konaklieva S., Popov S. (2006). Volatile compounds from some Black Sea red algae and their chemotaxonomic application. Bot. Mar..

[31] Ludwiczuk A., Nagashima F., Gradstein R.S., Asakawa Y. (2008). Volatile components from selected Mexican, Ecuadorian, Greek, German and Japanese liverworts. Nat. Prod. Commun..

[32] Ji X.M., Bossé Y., Landi M.T., Gui J., Xiao X.J., Qian D., Joubert P., Lamontagne M., Li Y.F., Gorlov I. (2018). Identification of susceptibility pathways for the role of chromosome 15q25.1 in modifying lung cancer risk. Nat. Commun..

[33] El-Shaibany A., Al-Habori M., Al-Maqtari T., Al-Mahbashi H. (2020). The yemeni brown algae *Dictyota dichotoma* exhibit high in vitro anticancer activity independent of its antioxidant capability. Biomed Res. Int..

[34] Adkins D.E., Khachane A.N., McClay J.L., Åberg K., Bukszár J., Sullivan P.F., van den Oord E.J.C.G. (2012). SNP-based analysis of neuroactive ligand–receptor interaction pathways implicates PGE2 as a novel mediator of antipsychotic treatment response: Data from the CATIE study. Schizophr. Res..

[35] NIST Chemistry WebBook, SRD 69. https://webbook.nist.gov/chemistry/.

[36] Lab of System Pharmacology. https://tcmspw.com/tcmsp.php.

[37] Unitprot. https://www.uniprot.org/.

[38] STRING. https://string-db.org/.

[39] DAVID Bioinformatics Resources 6.8. https://david.ncifcrf.gov/.

