# Supplementary material for: Characteristic Volatile Composition of Seven Seaweeds from the Yellow Sea of China
Source: Mar Drugs. 2021 Mar 29;19(4):192. doi: 10.3390/md19040192 (PMC8066643; doi:10.3390/md19040192)
Supplement: Supplementary file 1 [file marinedrugs-19-00192-s001.pdf]

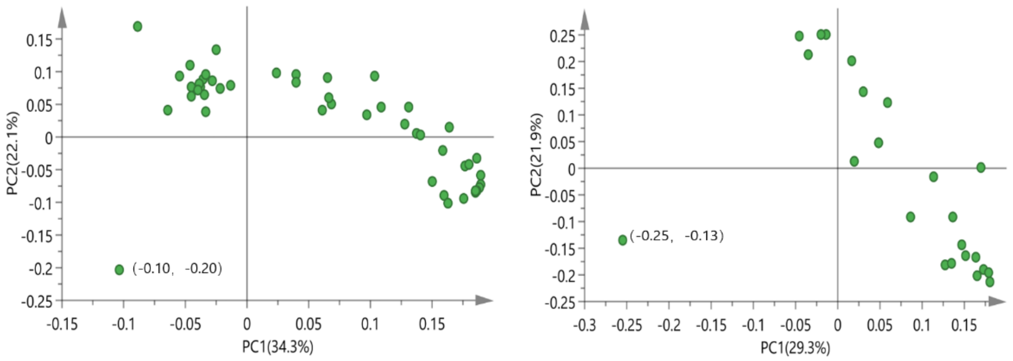

(**a**) (**b**)

Figure S1. PCA of VOCs compositions in seven seaweeds by HS-SPME-GC-MS, loading plot with: (**a**) DVB/CAR/PDMS fiber; (**b**) PDMS fiber.

Table S1. The compounds corresponding to dot with coordinates in loading plot

coordinates	(-0.10, -0.20)	(-0.25, -0.13)
Compound	β-Bourbonene	β-Bourbonene
	γ-Muurolene	γ-Muurolene
	Germacrene D	Germacrene D
	cis-Muurola-3,5-diene	α-Selinene
	β-Gurjunene	β-Cadinene
	Cubenol	Cubenol
	Muurola-4,9-diene	Thunbergol
	β-Copaene	Geranyl-α-terpinene
	1,4-Cadinadiene	1,5,9-Trimethyl-12-(1-methylethyl)-4,8,13-Cyclotetradecatriene-1,3-diol
	α-Muurolene	Cedrene
	(E)-2-Hepten-1-ol	
	α-Cubebene	
	δ-Cadinene	
